# Revolutionizing attention deficit hyperactivity disorder with artificial intelligence

**DOI:** 10.1371/journal.pmen.0000181

**Published:** 2024-11-25

**Authors:** Archana Reddy Bongurala, Dhaval Save, Ankit Virmani

**Affiliations:** 1 Department of Pediatrics, Omni Family Health, Bakersfield, California, United States of America; 2 Department of Internal Medicine, Methodist Medical Center of Illinois, Peoria, Illinois, United States of America; 3 Department of Artificial Intelligence, Virufy Inc., Los Altos, California, United States of America; PLOS: Public Library of Science, UNITED KINGDOM OF GREAT BRITAIN AND NORTHERN IRELAND

Attention-deficit/hyperactivity disorder (ADHD) is a prevalent neurodevelopmental disorder affecting millions of individuals worldwide, posing significant challenges in accurate diagnosis and effective management [1]. ADHD impacts 3–10% of children, causing inattention, hyperactivity, and impulsivity that disrupt social, academic, and occupational life, potentially lasting into adulthood [2]. The complex symptomatology and heterogeneous nature of ADHD have long posed difficulties for healthcare professionals, leading to misdiagnosis, delayed management, and suboptimal outcomes. However, the rapid advancements in artificial intelligence (AI) offer a transformative opportunity to revolutionize the diagnosis and management of ADHD, providing a more objective, data-driven approach to improve patient outcomes.

## Enhancing diagnostic accuracy

Traditional methods of diagnosis of ADHD in pediatric and adolescent individuals relies on a comprehensive approach encompassing a detailed clinical history, physical examination, review of information from home and community settings, and application of the Diagnostic and Statistical Manual of Mental Disorders, Fifth Edition (DSM-5) criteria [3]. Reliance on subjective reports can be biased and miss co-existing conditions. The thorough evaluation process can be time-consuming and resource-heavy. Additionally, standardized assessments might not capture cultural nuances, potentially leading to misdiagnosis. These limitations pave the way for AI to potentially improve ADHD diagnosis, accuracy and efficiency [3].

**Fig 1:**
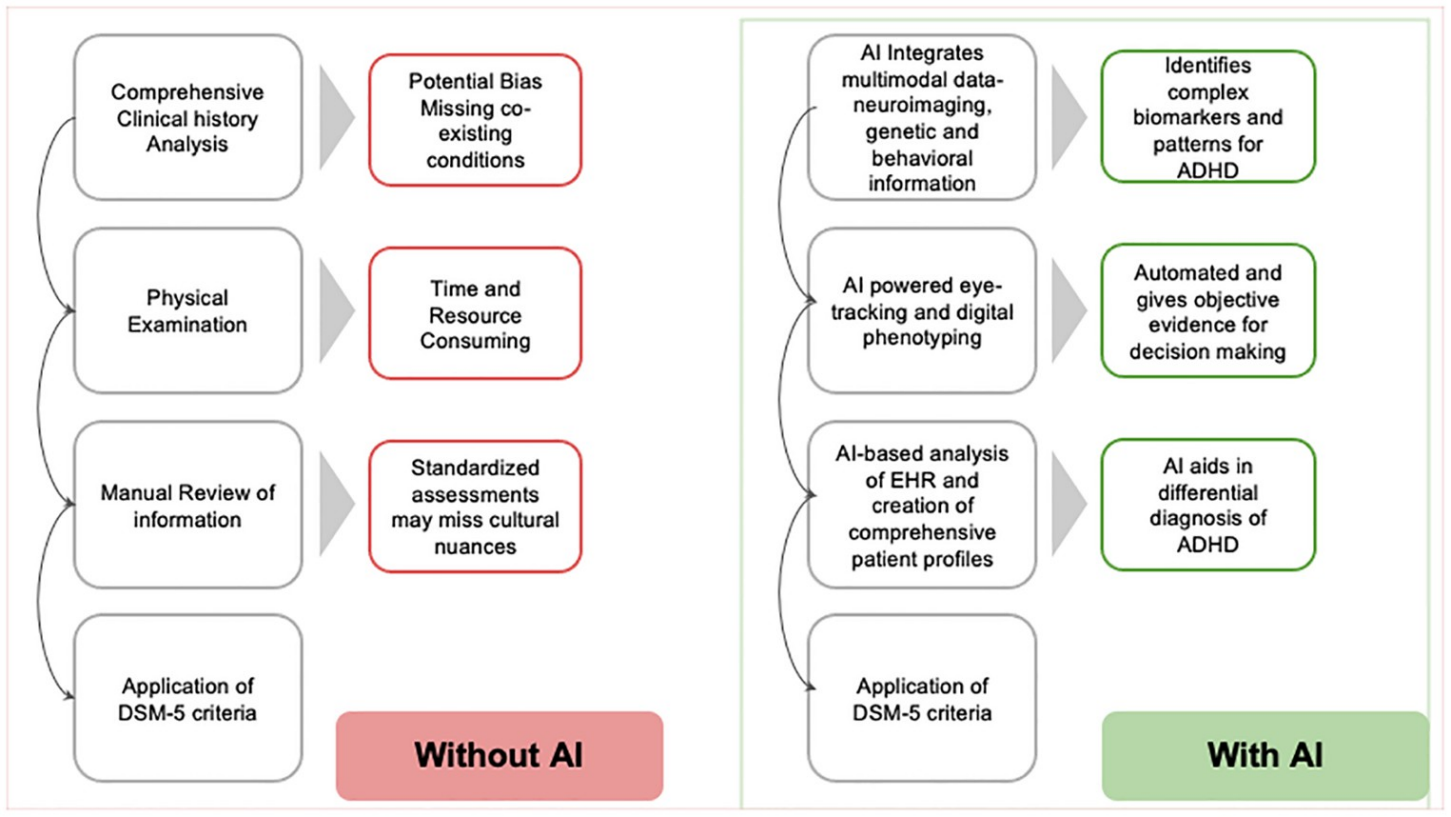
Impact of AI on diagnosis and management of ADHD.

As shown in Fig 1, AI’s ability to process and analyze vast amounts of multimodal data, including neuroimaging, genetic, and behavioral information, has the potential to identify complex patterns and biomarkers associated with ADHD. Machine learning algorithms, such as support vector machines and deep neural networks, have shown promising results in classifying ADHD based on functional magnetic resonance imaging (fMRI) data, achieving high accuracy rates [4]. These AI-based approaches can detect subtle abnormalities in brain connectivity patterns that may be overlooked by human observers, providing objective evidence to support clinical decision-making. Moreover, AI can integrate multiple data sources, such as neuropsychological assessments, electronic health records, and even digital phenotyping data from wearable devices, to create comprehensive patient profiles and aid in the differential diagnosis of ADHD [5].

Recent studies have explored innovative AI-based approaches to enhance ADHD screening and diagnosis. For instance, Yoo et al. [2] developed a machine learning model using portable eye tracking data to assist in ADHD screening. The study demonstrated the potential of eye movement patterns as biomarkers for ADHD, with the AI model achieving promising accuracy in distinguishing between individuals with and without ADHD. Such AI-powered screening tools could provide a more accessible and cost-effective means of early identification, enabling timely intervention and support, as long as they are used in conjunction with other sources of information.

### Personalizing treatment approaches

Attention-deficit/hyperactivity disorder (ADHD) treatment in children and adolescents utilizes a multimodal approach, combining behavior modification therapy with medication. This strategy aims to improve core symptoms, optimize functional outcomes, and address behavioral impediments [3].

AI holds immense promise in personalizing approaches for helping individuals with ADHD to manage their symptoms. AI can predict individual responses to medications and therapies, enabling healthcare providers to customize treatment based on the person’s unique characteristics [6]. For example, AI algorithms can analyze genetic profile, clinical history, and previous treatment outcomes to identify the most effective medication and optimal dosage, minimizing the risk of adverse effects and improving treatment adherence [7]. Furthermore, AI can continuously monitor treatment progress through wearable devices and mobile applications, providing real-time feedback and enabling timely adjustments to optimize therapeutic outcomes.

Pharmacological interventions, particularly stimulant medications, are a cornerstone of ADHD treatment. However, response to medication varies widely among individuals, and finding the optimal medication and dosage often involves a trial-and-error process [6]. AI-powered precision medicine approaches can predict individual treatment responses, enabling healthcare providers to tailor medication regimens based on a patient’s unique characteristics [7].

AI can minimize the risk of adverse effects, and improve treatment adherence by identifying potential responders and non-responders.

### Empowering self-management

The integration of AI-powered tools, such as mobile applications and virtual assistants, can empower individuals with ADHD to better manage their condition in daily life. These technologies can offer personalized reminders, organize tasks, and provide real-time coaching to enhance self-management skills and executive functioning [8]. For instance, an AI-powered virtual coach can analyze an individual’s daily routines, provide tailored strategies to improve time management and reduce distractions, offering encouragement and support to maintain motivation. Moreover, AI can facilitate the delivery of cognitive-behavioral therapy (CBT) and other evidence-based interventions through interactive platforms, increasing access to effective treatments for individuals who may face barriers to traditional in-person therapy [8].

Berrezueta-Guzman et al. [1] evaluated the efficacy of using ChatGPT, an AI-powered large language model, in enhancing therapy for ADHD. The study found that ChatGPT could generate personalized therapy content, provide real-time feedback, and engage patients in interactive conversations, potentially improving results. The integration of AI-based virtual assistants like ChatGPT into ADHD care could offer a scalable and accessible means of delivering personalized support and therapy, particularly in resource-limited settings.

### Considerations and limitations

The responsible implementation of AI in ADHD diagnosis and management is crucial to ensure ethical, transparent, and equitable outcomes. Data privacy and security measures should be established to protect patient information, and algorithmic bias must be actively mitigated to prevent perpetuating existing healthcare disparities. Collaboration among healthcare professionals, AI experts, and community advocates is essential to develop and validate AI-based tools that are clinically relevant, user-friendly, and aligned with individual needs and values.

It is essential to recognize that some individuals with ADHD view certain traits as strengths and may not seek to ’manage’ them in conventional ways. AI has the potential to develop personalized interventions that account for both environmental influences and individual strengths, offering tailored support for children who may need additional assistance.

## Conclusion

In conclusion, AI holds immense promise in ADHD diagnosis and management by providing objective, data-driven insights and personalized treatment approaches. By harnessing the power of machine learning and multimodal data integration, AI can enhance diagnostic accuracy, optimize medication management, and deliver personalized non-pharmacological interventions. However, the responsible development and deployment of AI-based tools, with a focus on ethical considerations and person-centered care, are crucial to fully realize AI’s potential in improving the lives of individuals with ADHD.

As we deepen our understanding of the interconnectedness between various mental health conditions, ADHD may no longer be viewed through such a narrow lens. This shift could lead to more holistic, transdiagnostic interventions. AI has the potential to drive this change by reducing cultural and gender biases, offering more equitable and personalized treatments.
